# Clinicopathological Characteristics and Survival Analysis of Primary Mesenteric Liposarcoma: A Retrospective Study

**DOI:** 10.3390/medicina61111984

**Published:** 2025-11-05

**Authors:** Zeki Ogut, Adem Tuncer, Yasin Dalda, Harika Gozde Gozukara Bag, Mehmet Bugra Bozan

**Affiliations:** 1 Department of Surgery, Elazig Fethi Sekin City Hospital, 23300 Elazig, Turkey; 2 Department of Surgery, Liver Transplant Institute, Inonu University Faculty of Medicine, 44280 Malatya, Turkey; 3 Department of Biostatistics, Inonu University Faculty of Medicine, 44280 Malatya, Turkey

**Keywords:** primary mesenteric liposarcoma, survival analysis, dedifferentiated liposarcoma, surgical outcomes, recurrence, prognosis

## Abstract

*Background and Objectives:* Primary mesenteric liposarcoma (LPS) is an exceptionally rare malignancy, with most literature data limited to isolated case reports or small series. This papers aims to evaluate the clinicopathological features, treatment outcomes, and prognostic factors in patients with mesenteric LPS. *Materials and Methods:* Thirteen patients diagnosed with primary mesenteric LPS between 2010 and 2022 were retrospectively analyzed. Data included demographics, tumor location, histological subtype, surgical treatment, recurrence, and survival. *Results:* The median age was 56 years (range, 22–74), with a slight male predominance (53.8%). Most tumors arose from the small bowel (53.8%) and colonic (38.5%) mesenteries, with one involving the gastric mesentery. The predominant histological subtypes were myxoid (46.1%) and dedifferentiated (23.1%). R0 resection was achieved in 76.9% of patients. During a median follow-up of 55.2 months, nine patients (69.2%) developed recurrence. Mortality was higher in patients with dedifferentiated LPS (66.7%) than in those with myxoid LPS (40%). Five-year survival rate was 100% in patients without recurrence and 28.6% in those with recurrence (*p* = 0.112, not significant). Patients who received adjuvant chemoradiotherapy suggested longer survival (110.7 vs. 46.2 months; *p* = 0.620). *Conclusions:* This 12-year study highlights the aggressive nature of mesenteric LPS, particularly the dedifferentiated subtype which showed the poorest prognosis. Complete resection remains the primary treatment; however, it has high recurrence rates. To diminish the catastrophic poor results of the postoperative period, multidisciplinary treatment strategies become a keystone.

## 1. Introduction

Liposarcoma (LPS) is the most prevalent type of soft tissue sarcoma in adults, typically appearing in individuals in their fifties or sixties, with a slight predilection for males [1,2]. Although LPS primarily develops in the deep soft tissues of the limbs and retroperitoneum, it can also occur less frequently in the neck, mediastinum, trunk, and groin [3]. The World Health Organization (WHO) classifies LPS into four histological types: well-differentiated, dedifferentiated, myxoid, and pleomorphic [4]. Each subtype exhibits distinct morphological and biological behaviors. Well-differentiated tumors tend to have indolent growth, whereas dedifferentiated and pleomorphic variants show more aggressive clinical courses, and myxoid LPS demonstrates an intermediate prognosis. Retroperitoneal and intra-abdominal LPSs often remain asymptomatic until they reach a considerable size, with symptoms typically manifesting based on tumor location or secondary complications [5,6]. This delay in symptom onset often results in large tumor volumes at the time of diagnosis and increases the risk of incomplete resection.

Primary mesenteric LPS is an exceedingly rare condition, with most data derived from isolated case reports or very small series [7]. The scarcity of published data leads to a limited understanding of its clinicopathological features, treatment strategies, and long-term outcomes. Compared with retroperitoneal LPSs, mesenteric tumors pose distinct diagnostic and therapeutic challenges, particularly because of nonspecific symptoms and close proximity to major mesenteric vessels. Non-specific symptoms, such as abdominal swelling, pain, or bowel obstruction, often lead to delayed diagnosis. Radiological findings may resemble those of other mesenteric tumors or cystic lesions, complicating preoperative assessment. Additionally, surgical management can be technically demanding because of the proximity of an LPS to vital mesenteric vessels and the frequent need for bowel resection to achieve complete tumor resection.

Histological subtype is a key prognostic factor. Well-differentiated tumors generally progress slowly, and dedifferentiated and pleomorphic variants are associated with high recurrence and poor survival rates. Myxoid LPSs, though less aggressive, still carry a significant risk of local and systemic relapse. Consequently, the biological behavior of mesenteric LPS remains unpredictable, and therapeutic strategies remain poorly defined.

This study aimed to investigate the clinicopathological characteristics, treatment outcomes, and prognostic factors of patients with primary mesenteric liposarcoma, in order to provide additional long-term evidence and contribute to the limited body of literature on this rare entity.

## 2. Materials and Methods

### 2.1. Study Design and Patient Selection

Inclusion criteria were as follows: age of at least 18 years old at the time of diagnosis; have histopathological confirmation of primary mesenteric LPS; and tumor originating solely in the mesentery, with no evidence of retroperitoneal origin or secondary mesenteric involvement. The study was conducted as a retrospective, observational analysis between 2010 and 2022. Comprehensive clinical, pathological, treatment, and follow-up data were also available.

Exclusion criteria were retroperitoneal LPS or tumors of uncertain origin extending into the mesentery; secondary mesenteric involvement due to another primary cancer; and incomplete demographic, clinicopathological, or follow-up information.

### 2.2. Data Collection

Demographic characteristics (age and sex), tumor localization (small bowel, colon, or gastric mesentery), tumor size, histological subtype, surgical procedures, resection margin status (R0, R1, or R2), adjuvant treatments (radiotherapy and/or chemotherapy), recurrence status, and survival outcomes were extracted from patient records and pathology reports. Recurrence was defined as radiologically or pathologically confirmed reappearance of the tumor after curative surgery. Overall survival was calculated from the date of surgery to death or last follow-up.

### 2.3. Histopathological Evaluation

Histopathological diagnoses were established according to the World Health Organization (WHO) Classification of Soft Tissue and Bone Tumors valid at the time of diagnosis (2013 or 2020 editions). All cases were reviewed by experienced pathologists to ensure consistency with the 2020 terminology and grading system. Immunohistochemical (IHC) staining included MDM2, CDK4, S100, desmin, and smooth muscle actin (SMA) to confirm the diagnosis and differentiate subtypes. Molecular amplification testing for MDM2 and CDK4 was performed in selected cases when histological findings were inconclusive.

### 2.4. Adjuvant Treatment

Chemotherapy regimens consisted mainly of doxorubicin with or without ifosfamide. Radiotherapy was delivered with a total dose ranging from 45 Gy to 60 Gy in fractions of 1.8–2.0 Gy. Two patients received combined chemoradiotherapy, while others underwent single-modality adjuvant therapy based on multidisciplinary tumor board recommendations.

### 2.5. Follow-Up Protocol

During the first 2 years, the patients underwent physical examinations and blood tests every 3 months for monitoring. Abdominal imaging (ultrasound or computed tomography) was performed every 6 months or sooner if clinically indicated. Disease recurrence and survival status were recorded at each visit. Follow-up data were censored at the last known contact if the patient was alive. Thereafter, follow-up intervals were individualized according to recurrence risk and disease progression.

### 2.6. Statistical Analysis

The parametric variables are presented as medians and ranges (minimum–maximum). The Shapiro–Wilk test was used to assess the normality of distribution for parametric variables. Depending on the results, parametric variables were compared using Mann–Whitney U test. The parametric values were given as median. For categorical variables, comparisons were made using Fisher’s exact test, depending on which test was more suitable. The categorical values were given as count (n) and percentage (%). The Kaplan–Meier method was used for survival analyses, and subgroup comparisons were performed using the log-rank test. Statistical analyses were conducted using IBM SPSS Statistics for Windows (version 25.0; IBM Corp., Armonk, NY, USA), with a *p*-value of <0.05 considered statistically significant.

### 2.7. Ethical Approval

The Non-Interventional Clinical Research Ethics Committee at the Faculty of Medicine, İnönü University approved this study (Approval No. 2024/6869), which was conducted in accordance with the Declaration of Helsinki.

The language of this manuscript has been revised using ChatGPT(GPT-5, OpenAI, San Francisco, CA, USA), an artificial intelligent-based language model developed by OpenAI.

## 3. Results

### 3.1. Patient and Tumor Characteristics

Between 2010 and 2022, a total of 13 patients (6 males, 7 females) with primary mesenteric liposarcoma (LPS) were managed at our institution; 12 underwent surgical resection, and one was diagnosed by biopsy without surgery. The median age was 56 years (range, 22–74). The most common tumor locations were the small bowel mesentery (n = 7, 53.8%) and colonic mesentery (n = 5, 38.5%), with one case involving the gastric mesentery. Table 1 summarizes patients’ clinicopathological characteristics.

Most tumors were located in the small bowel (n = 7, 53.8%) and colonic (n = 5, 38.4%) mesenteries, with one involving the gastric mesentery. Median tumor size was 19 cm, with sizes between 5.5–35 cm. The predominant histological subtypes were myxoid (n = 6, 46.1%) and dedifferentiated (n = 3, 23.1%), followed by well-differentiated (n = 3, 23.1%) and pleomorphic (n = 1, 7.7%).

**Figure 1:**
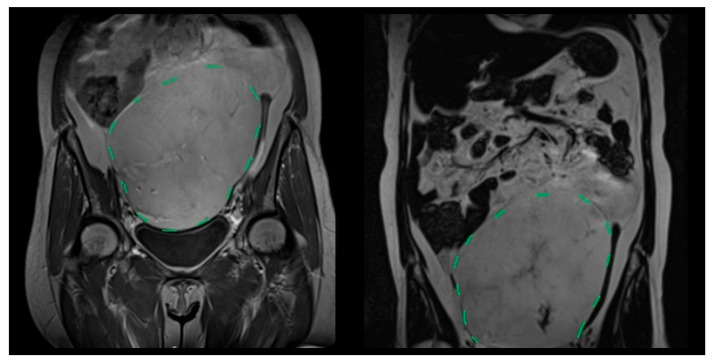
Coronal T2-weighted MRI showing a giant primary mesenteric liposarcoma extending from the pelvis to the upper abdomen (green dashed line indicates the tumor boundary).

**Figure 2:**
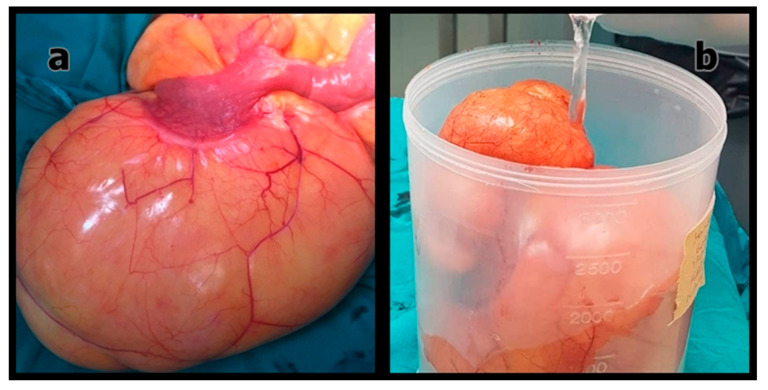
(**a**) Intraoperative appearance of the primary mesenteric liposarcoma; (**b**) Macroscopic view of the resected tumor after excision.

**Figure 3:**
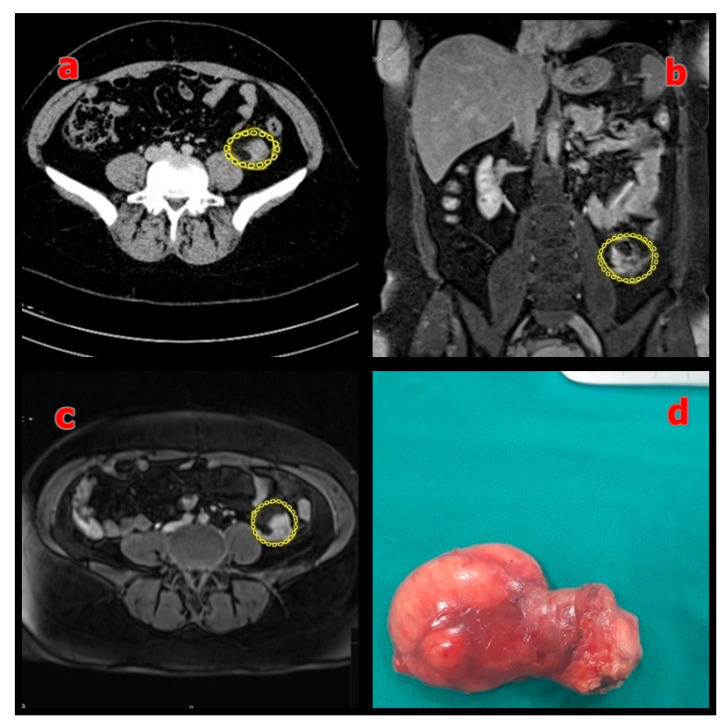
(**a**) Preoperative axial MRI showing the mesenteric mass (sigmoid colon mesentery) outlined in yellow; (**b**) Coronal MRI showing the same mass outlined in yellow; (**c**) Axial T2-weighted MRI confirming the lesion (outlined in yellow); (**d**) Macroscopic view of the resected specimen with negative surgical margins.

Figure 1, Figure 2 and Figure 3 show representative radiological and intraoperative findings.

### 3.2. Surgical and Oncological Outcomes

Of the 12 patients who underwent surgery, R0 resection was achieved in 10 (83.3%), and R1 in 2 (16.7%): no R2 resections were performed. One patient underwent biopsy alone and did not undergo surgical resection. Median follow-up duration was 55 months between 0.4–158 months. During a median follow-up of 55.2 months (range, 0.4–158), nine patients (69.2%) developed local or systemic recurrence, consistent with recurrence rates reported for intra-abdominal LPS. Table 2 details the distribution of recurrence and mortality in relation to clinicopathological parameters.

At the last follow-up, six (46.1%) patients died, while seven (53.9%) were still alive. Notably, both patients who received combined adjuvant chemoradiotherapy remained free of recurrence, whereas three of four patients treated with chemotherapy alone died. Based on histological subtype analysis, mortality rate was 40% for myxoid LPS and 66.7% for dedifferentiated LPS.

Recurrence was more frequently observed in tumors originating from the small bowel or colonic mesentery (seven of nine patients, 77.8%) than that originating from other mesenteric sites (0 of two patients, 0%), although this difference was not statistically significant (*p* = 0.109). Similarly, recurrence occurred in all patients with tumors <15 cm (2/2, 100%) and in 55.6% of those with tumors ≥15 cm (5/9), without statistical significance (*p* = 0.491).

### 3.3. Survival Analysis

Table 3 presents the Kaplan–Meier survival analysis for recurrence and adjuvant treatment. For patients without recurrence, median overall survival (OS) was not reached, whereas it was 28.6 months (range, 6–74 months) for those with recurrence (*p* = 0.112). Two patients were excluded from survival analysis due to incomplete follow-up. Patients who received adjuvant therapy (chemotherapy and/or radiotherapy) had a median survival of 158 months, compared with not reached (NR) in those without adjuvant treatment (*p* = 0.620). None of the evaluated clinical parameters had a significant effect on recurrence or survival (all *p* > 0.05).

Kaplan–Meier survival curves were updated with higher resolution and labeled as “Adjuvant (+)” and “Adjuvant (–)” groups for clarity. A detailed summary of adjuvant therapy modalities and outcomes is provided in Table 4.

## 4. Discussion

In this 13-patient cohort, the primary mesenteric LPSs were generally large at presentation, with a median size of 19 cm, and most of them were myxoid or dedifferentiated (38.4% each). Recurrence was observed in 69.2% of the patients and was closely associated with poor outcomes, as five-year survival reached 100% in those without recurrence, but decreased to 28.6% in those with recurrence. These findings suggest an aggressive clinical behavior of mesenteric LPSs, even when complete surgical removal appears to be achieved in most patients.

Primary mesenteric LPS is an extremely uncommon tumor, with most of the understanding of its clinicopathological features and prognosis derived from case studies and limited series [8,9,10]. Similarly to retroperitoneal LPS, mesenteric LPSs tends to grow slowly and remain asymptomatic until they reach a considerable size, often leading to late detection and diagnosis at advanced stages [5,6]. A combination of delayed symptom onset and tumor rarity complicates the process of diagnosis and establishment of standardized treatment guidelines. This gap underscores the importance of reporting institutional experiences to expand current understanding.

In our study group, the average age at diagnosis was 48.5 years, which was lower than that reported by Rosato and Hasegawa (59 and 60 years, respectively) [11,12]. However, the male predominance was consistent with previous findings. Dedifferentiated LPS strictly limited to the mesentery is extremely rare, highlighting the importance of our observations [13]. These subtypes are known for their higher recurrence potential and variable responses to adjuvant therapy, explaining the heterogeneity in survival outcomes reported across studies. Histologically, myxoid and dedifferentiated subtypes were the most common, accounting for 38.4% of patients, which is consistent with the findings of previous studies [12,14].

At diagnosis, mesenteric LPS tumors can be large, typically measuring 12–32 cm, as observed in our series, reflecting a tendency for late detection [15,16]. Complete surgical resection with clear margins remains the primary treatment approach. In our cohort, R0 resection was achieved in 76.9% of the patients, while two had microscopic positive margins (R1, 16.7%). The significance of surgical margin status is well recognized in retroperitoneal sarcomas [17] and appears equally critical in mesenteric cases, emphasizing the need for careful surgical planning.

Recurrence is the main challenge in the management of this condition. Although a retroperitoneal series reported recurrence rates of approximately 41.4% [12], our study found a recurrence rate of 69.2%, suggesting a potentially more aggressive behavior in mesenteric tumors. Jain et al. reported a case of mesenteric LPS with negative margins and no nodal involvement that recurred after 26 months [18], illustrating the unpredictable nature of this disease. Other studies on gastrointestinal and mesenteric LPS also highlight the rarity of this condition and difficulties in establishing prognostic patterns [19]. This observation, together with prior case reports, suggests that mesenteric localization may confer a higher risk of recurrence than retroperitoneal disease.

In patients without recurrence, the 5-year survival rate was 100%. Conversely, the rate decreased to 28.6% in patients who experienced recurrence. Although this difference was not statistically significant (*p* = 0.112), its clinical importance was evident, and the histological subtype appeared to influence prognosis. Patients with dedifferentiated LPS had a higher mortality rate (66.7%) than those with myxoid LPS (40%), which is consistent with previous studies characterizing dedifferentiated tumors as biologically aggressive and associated with poor survival outcomes [20,21,22]. This pattern reinforces the necessity of subtype-specific management and closer follow-up for dedifferentiated variants. As previously reported, dedifferentiated LPS not only has a higher likelihood of local recurrence but also poses a significant risk of distant metastasis, occurring in approximately 30% of patients [23]. This highlights the need for more stringent monitoring strategies in patients with dedifferentiated diseases.

However, the efficacy of adjuvant therapy remains controversial. In our study, patients who received combined chemoradiotherapy tended to have better survival outcomes than those who received chemotherapy alone or no adjuvant therapy, although the difference was not statistically significant. This apparent difference may partly reflect confounding by indication, as adjuvant therapy was preferentially offered to higher-risk patients. Data from retroperitoneal sarcoma cohorts suggest that radiotherapy can improve local control; however, its effect on overall survival is limited [24,25,26]. Furthermore, extensive studies on advanced dedifferentiated LPS indicates that chemotherapy has limited effectiveness, with low response rates and median progression-free and overall survival times of just 4.6 and 15.2 months, respectively [27]. Collectively, these findings highlight the urgent need for effective systemic treatments. Recent data also highlight CDK4 amplification as a prognostic and therapeutic biomarker in dedifferentiated liposarcoma, offering potential for targeted therapy [28].

Recent studies indicated that immune and molecular characteristics can enhance risk assessment. Zhao et al. discovered that the immune microenvironment, particularly the presence of regulatory T and CD8+ T cells, appears strongly associated with survival in dedifferentiated subtypes [29]. Thus, the integration of molecular and immunological profiling may offer a more personalized approach to postoperative care and patient selection for additional therapies.

Limitations: Although this study provides valuable insights, it has several limitations. First of all, it is a retrospective and single-center experience study. Additionally, the small sample size inherent to the rarity of primary mesenteric liposarcoma, inevitably limits the statistical power and generalizability of the findings. In addition, the heterogeneity of adjuvant therapy protocols and potential confounding by indication may have influenced survival outcomes.

Selection bias and incomplete follow-up data could also have affected the accuracy of recurrence and survival analyses. Furthermore, the lack of consistent molecular confirmation (e.g., MDM2/CDK4 amplification testing) restricts the ability to draw definitive biological conclusions.

Despite these constraints, this study represents one of the largest long-term institutional experiences reported to date and provides valuable quantitative insights into recurrence patterns and survival outcomes in primary mesenteric liposarcoma. These limitations should be considered when interpreting the apparent association between adjuvant therapy and improved survival. In summary, mesenteric LPSs are rare, slow-growing tumors that often present at an advanced stage with vague symptoms. Dedifferentiated subtypes exhibited aggressive progression, with high recurrence and mortality rates. These results emphasize the importance of vigilant postoperative monitoring, multidisciplinary care, and future multicenter prospective studies incorporating molecular and immunological profiling to better define optimal treatment strategies for this rare tumor type.

## 5. Conclusions

This study constitutes one of the most extensive single-institution collections of primary mesenteric LPSs documented to date, and provides valuable insights into their clinicopathological characteristics and survival rates. Our findings underscore the particularly aggressive nature of dedifferentiated subtypes, which are associated with notably higher mortality rates than other histological types. Although complete surgical excision remains the primary treatment strategy, the high recurrence rate highlights the importance of vigilant, postoperative monitoring. Although combined chemoradiotherapy has the potential to enhance survival, its efficacy remains uncertain and warrants validation in larger studies. Given the rarity of mesenteric LPSs and limited evidence available, multicenter prospective studies incorporating molecular and immunological profiling are needed to better define optimal therapeutic strategies and enhance clinical understanding and patient outcomes.

## Figures and Tables

**Table 1:** Clinicopathological features of patients with primary mesenteric liposarcoma.

Patient No	Age	Sex	Tumor Location	Histology	Tumor Size(cm)	RT/CT	Recurrence	Mortality	Follow-Up Duration (Months)
Patient 1	55	F	Jejunal	Well-differentiated	20 × 15 × 12	No	No	Alive	76.4
Patient 2	33	F	Sigmoid colon mesentery	Well-differentiated	5.5 × 3.5 × 2.5	RT	Yes	Alive	85.7
Patient 3	57	M	Small bowel mesentery	Dedifferentiated	19 × 15 × 13	CT	Yes	Deceased	158.0
Patient 4	69	F	Left colon	Dedifferentiated	32 × 29 × 22	No	Yes	Deceased	0.4
Patient 5	64	F	Stomach	Pleomorphic Liposarcoma	18 × 15 × 10	-	-	Deceased	34.4
Patient 6	74	M	Small bowel mesentery	Myxoid Liposarcoma	28 × 19 × 13	CT	Yes	Deceased	45.4
Patient 7	43	F	Left colon	Myxoid Liposarcoma	12 × 11 × 2	RT-CT	Yes	Alive	117.2
Patient 8	42	M	Jejunoileal and Colonic Mesentery	Myxoid Liposarcoma	16 × 11 × 3	No	No	Deceased	1.4
Patient 9	66	M	Right Upper-Lower Quadrant	Well-differentiated	26 × 21 × 28 (FNA biopsy)	No	- *	Deceased (metastatic disease)	-
Patient 10	40	M	Left Lower Quadrant	Myxoid Liposarcoma	17 × 14 × 11.5	No	No	Alive	71.8
Patient 11	22	F	Right Colon	Myxoid Liposarcoma	23 × 18.5 × 15	No	-	Alive	35.7
Patient 12	71	M	Jejunoileal and Colonic Mesentery	Myxoid Liposarcoma	19 × 19 × 14	CT	-	Deceased	14.8
Patient 13	56	M	From Pelvis to Right Hepatic Lobemesentery	Dedifferentiated	35 × 20 × 10	No	-	Alive	35.8

Abbreviations: F—female; M—male; RT—radiotherapy; CT—chemotherapy. *—One patient underwent biopsy only and did not undergo surgical resection.

**Table 2:** Distribution of recurrence and mortality according to clinical parameters.

Parameter	Category	No Recurrence n (%)	Recurrence n (%)	*p*	Alive n (%)	Deceased n (%)	*p*
Tumor Location	Colon + SB-mesentery	2 (22.2)	7 (77.8)		4 (44.4)	5 (55.6)	1.000
	Other mesenteric are	2 (100)	0 (0)	0.109	2 (50)	2 (50)	
Tumor Size	<15 cm	0 (0)	2 (100)	0.491	2 (100)	0 (0)	0.192
	≥15 cm	4 (44.4)	5 (55.6)		4 (36.4)	7 (63.6)	
Histological Subtype	Well-differentiated	1 (50)	1 (50)	1.000	2 (66.7)	1 (33.3)	1.000
	Dedifferentiated	1 (33.3)	2 (66.7)		1 (33.3)	2 (66.7)	
	Myxoid	2 (33.3)	4 (66.7)		3 (42.9)	4 (57.1)	
RT/CT Received	No	—	—	—	3 (50)	3 (50)	1.000
	Yes	—	—	—	3 (50)	3 (50)	
Recurrence Status	No	—	—	—	4 (100)	0 (0)	0.061
	Yes	—	—	—	2 (28.6)	5 (71.4)	

Abbreviations: SB—small bowel; RT—radiotherapy; CT—chemotherapy. Other mesenteric areas include stomach mesentery and unspecified mesenteric sites.

**Table 3:** Survival analysis according to recurrence status and adjuvant therapy (Kaplan–Meier method).

**A. Case Processing Summary**				
	**Total N**	**Number of Events**	**Censored n (%)**	***p***
Recurrence				0.112
Yes	7	5	2 (28.6%)	
No	4	0	4 (100.0%)	
Overall	11	5	6 (54.5%)	
RT/CT				0.620
Yes	6	3	3 (50.0%)	
No	5	2	3 (60.0%)	
Overall	11	5	6 (54.5%)	
**B. Mean and Median Survival Times According to Recurrence and Adjuvant Therapy**				
**Parameter**	**Survival Time** **(Median, Months)**	**Std. Error**	**95% CI Lower**	**95% CI Upper**
Recurrence: Yes	28.6	8.3	12.5	44.7
Recurrence: No	Not reached	—	—	—
RT/CT: Yes	110.7	32.843	46.302	175.048
RT/CT: No	46.2	16.542	13.778	78.622

Abbreviations: CI—confidence interval; RT—radiotherapy; CT—chemotherapy. Note: Median survival was not reached in the RT/CT: No and Recurrence: No groups due to insufficient number of events or complete censoring.

**Table 4:** Summary of adjuvant therapies and clinical outcomes in patients with primary mesenteric liposarcoma.

Adjuvant Therapy Type	n	Recurrence (n, %)	Deceased (n, %)	Median Survival (months)	Comments
None	5	3 (60%)	3 (60%)	46	Baseline group: poorer survival.
Chemotherapy only	4	3 (75%)	3 (75%)	45	Limited benefit observed.
Radiotherapy only	1	0 (0%)	0 (0%)	85	Long-term disease control.
Combined RT + CT	2	0 (0%)	0 (0%)	117	Longest recurrence-free survival.

This table summarizes the adjuvant treatment modalities administered, including chemotherapy, radiotherapy, and combined chemoradiotherapy (CRT), together with recurrence status, survival outcomes, and median follow-up durations. Combined CRT was associated with the longest recurrence-free survival, although the difference was not statistically significant due to the limited sample size. Abbreviations: RT—radiotherapy; CT—chemotherapy; CRT—combined chemoradiotherapy; Gy—Gray; NA—not available.

## Data Availability

The datasets collected and analyzed during the current study are available from the corresponding author upon reasonable request.

## Abbreviations

The following abbreviations are used in this manuscript:

LPS	Liposarcoma
